# Sole adjuvant intraoperative breast radiotherapy in Taiwan: a single-center experience

**DOI:** 10.1186/s13058-021-01421-y

**Published:** 2021-04-01

**Authors:** Hsin-Yi Yang, Chi-Wen Tu, Chien-Chin Chen, Cheng-Yen Lee, Yu-Chen Hsu

**Affiliations:** 1 Clinical Medicine Research Center, Ditmansion Medical Foundation Chia-Yi Christian Hospital, Chia-Yi City, Taiwan 60002; 2 Department of Surgery, Ditmansion Medical Foundation Chia-Yi Christian Hospital, No. 539, Zhongxiao Rd., East District, Chia-Yi City, Taiwan 60002; 3 Department of Pathology, Ditmanson Medical Foundation Chia-Yi Christian Hospital, Chia-Yi City, Taiwan 60002; 4 Department of Cosmetic Science, Chia Nan University of Pharmacy and Science, Tainan, Taiwan 71710; 5 Department of Radiation Therapy and Oncology, Ditmanson Medical Foundation Chia-Yi Christian Hospital, Chia-Yi City, Taiwan 60002

**Keywords:** Breast cancer, Intraoperative radiotherapy, Whole breast external beam radiotherapy

## Abstract

**Introduction:**

Intraoperative radiotherapy (IORT) is more convenient than standard whole breast external beam radiotherapy (EBRT) as a sole adjuvant radiotherapy for breast cancer. The impact of age on breast cancer course and treatment strategy is still under investigation, and the peak age for breast cancer in Taiwan is much younger than that in Western countries. We aimed to review the oncological outcomes of sole IORT compared with standard EBRT in a country with younger breast cancer patients.

**Patients and methods:**

We reviewed patients with invasive breast cancer who received breast-conserving surgery (BCS) from September 2014 to December 2016. The clinicopathologic characteristics and oncological outcomes of eligible patients who received EBRT or IORT as sole adjuvant radiotherapy after BCS were collected and reviewed.

**Results:**

A total of 170 patients were enrolled with a mean follow-up time of 3.53 ± 0.82 years. The risk of locoregional recurrence was 2.44% for EBRT versus 10.64% for IORT (*p* = 0.024). IORT was a significant risk factor of locoregional recurrence (*p* = 0.005). The hazard ratios (HRs) for locoregional recurrence in the IORT group compared with the EBRT group were significantly higher in non-suitable risk group patients (HR = 7.02, *p* = 0.009) and in patients under 50 years old (HR = 10.42, *p* = 0.011).

**Conclusions:**

Locoregional recurrence was significantly higher in patients who received IORT than in those who underwent EBRT. IORT should not be used alone in patients under 50 years old who do not belong to a suitable group.

## Background

Adjuvant radiotherapy after breast-conserving surgery (BCS) halves the rate of the recurrence of 10-year disease and reduces the 15-year breast cancer death rate by about a sixth [1]. Standard whole breast external beam radiotherapy (EBRT) requires a lengthy treatment time of approximately 5–6 weeks, whereas intraoperative radiotherapy (IORT) offers convenient treatment once, concurrent with surgery. In addition to being more cost-effective, other advantages of IORT include precise brachytherapy of the target high-risk tissue and less heart–lung radiation dose exposure [2, 3]. The use of IORT as a sole adjuvant radiotherapy in the USA increased over 20-fold after the publication of the American Society for Radiation Oncology (ASTRO) accelerated partial breast irradiation (APBI) Consensus Guidelines and the targeted intraoperative radiotherapy versus whole breast radiotherapy for breast cancer (TARGIT-A) trial in 2010 [4]. A meta-analysis of 13 publications revealed that the breast cancer local recurrence rate after sole IORT was 0.02% per-person-month, with an adjusted 5-year recurrence rate of 2.7% [5]. These findings support the recent guidelines from the ASTRO supporting the use of sole IORT for low-risk patients.

The peak age for breast cancer is between 40 and 50 years in Taiwan, whereas the peak age in Western countries is between 60 and 70 years [6]. The age criteria for patients who are considered suitable for APBI radiotherapy were revised from older than 60 to 50 years in the updated 2016 ASTRO consensus. Whether age impacts the breast cancer course and treatment strategy remains a topic of interest. Indeed, in 2017, the Taiwan Intraoperative Radiotherapy Study Cooperative Group (T-IORTSCG) reported that patients who were selected for IORT in Taiwan tended to be younger, and the preliminary results were acceptable [7]. However, at present, there has been no comparison on the effect of sole IORT and standard EBRT in Taiwan. Therefore, the present study aimed to evaluate the difference in oncological outcomes between sole IORT and EBRT in a country with younger breast cancer patients.

## Methods/design

### Study design

From September 2014 to December 2016, 194 patients with invasive breast cancer who received BCS were reviewed. All the patients were treated in Chia-Yi Cristian Hospital (CYCH). After excluding 16 patients who refused radiotherapy and eight patients who had IORT with supplemental EBRT, 170 patients who received EBRT or IORT as a sole adjuvant radiotherapy were collected and reviewed. Clinicopathologic characteristics and oncological outcomes including patient characteristics, type of breast, and axillary surgery, tumor pathological results, type of adjuvant radiotherapy, type of concurrent treatment, type of recurrence, and survival status at the most recent follow-up were collected.

The inclusion criteria for IORT were unifocal invasive tumor of less than 3 cm, no evidence of lymph node involvement, and a minimum age of 40 years. These criteria were adapted from the T-IORTSCG study, the first leading multicenter study of IORT in Taiwan that was conducted by 11 Taiwanese hospitals with 9 medical centers included [7]. Radiation treatment options were explained to the patients who qualified for IORT, including standard EBRT, as well as IORT. All patients underwent extensive preoperative counseling from the surgeon. The protocol for conducting IORT via the Xoft Axxent eBx delivery system, in which the clinical effectiveness has been shown to be comparable with that of systems used in other IORT trials [8], is described by Hung-Wen Lai et al. in detail [7]. In our institute, intraoperative frozen sections for sentinel lymph node biopsies and margin status analysis were mandatory. After BCS, the tumor bed was mobilized to ensure that there was a distance of at least 10 mm between the surface of the applicator and the skin. A planned dose of 20 Gy to the balloon surface was delivered over 8 ± 15 min. After radiation treatment, the lumpectomy cavity was irrigated and closed in a standard manner. A positive resection margin was defined as positive tumor cells under microscopic exam. Patients were classified into different risk groups according to the ASTRO ABPI 2016 consensus [9]. Patients were considered as suitable for IORT if they fulfilled all the following criteria: Older than the age of 50, negative resection margin, negative axillary lymph node, tumor size ≦ 2.0 cm, negative for lymphovascular invasion, or positive hormone status. Patients were considered as unsuitable for IORT if they met any of the following criteria: Younger than the age of 40, positive resection margin, positive axillary lymph node, or tumor size > 3.0 cm. The other patients were categorized into a cautionary group. Locoregional recurrence, distant metastasis, and mortality were recorded and analyzed.

EBRT consisted of whole breast irradiation with regional lymph node irradiation reserved for high-risk patients. All treatment volumes were in accordance with the Radiation Therapy Oncology Group contouring atlas. The whole breast received either 40.5–42.6 Gy at 2.66 Gy per fraction or 50.0–50.4 Gy at 1.8–2.0 Gy per fraction. An additional 10–14 Gy was delivered to the tumor bed as a boost. The typical regional nodal irradiation included the ipsilateral axillary, supraclavicular, and internal mammary lymph nodes. All regional lymph nodes were treated with 50.4 Gy at 1.8 Gy per fraction. Regional lymph nodes were treated in all the patients with positive nodes. For the patients with negative sentinel nodes, regional nodal irradiation was prescribed to those with risk factors, such as grade III histology, ER negativity, lymphovascular invasion, and tumors measuring > 5 cm, with informed consent. The treatment planning goal was to cover at least 95% of the treatment volume with the prescribed dose. The major treatment planning constraint doses were 5 and 15 Gy to the whole heart and ipsilateral lung, and 45 Gy to the point dose in the spinal cord, respectively. Deep inspiratory breath-hold technique was prescribed to the patients with left breast cancer.

The follow-up protocol for the patients with breast cancer in CYCH includes the following: (1) clinical check-up every 3 months, (2) breast echocardiographic examination every 6 months, (3) yearly mammography, (4) yearly chest and abdominal computed tomography or chest radiography and abdominal echocardiographic examination, and (5) yearly bone scan examination. Any awareness of symptoms that indicate possible relapse or second tumors would be referred for further specialist consultation. The primary endpoints of this study were locoregional recurrence, overall survival, and breast cancer-specific survival. Patients in the cohort were followed until (1) locoregional recurrence; (2) death; (3) last contact if before the end of August 31, 2019; or (4) the end of August 31, 2019. Recurrence in the postoperative bed and/or ipsilateral regional lymph nodes was defined as a locoregional recurrence. The study was reviewed and approved by the Institutional Review Board of the Ditmanson Medical Foundation Chia-Yi Christian Hospital, Taiwan (CYCH-IRB no.: 2018009).

### Statistical analysis

Continuous variables were expressed as the mean ± standard deviation, and the categorical data were expressed as numbers and percentages. Continuous variables were compared using a *t* test, and categorical variables were compared using chi-squared test or Fisher’s exact test, as appropriate. Poisson regression was used to estimate the incidence rate (IR) ratios and their 95% confidence intervals (CIs) by comparing the IRs of different treatment methods with the IRs of the reference group. Kaplan–Meier analysis was used to measure the cumulative risks of locoregional recurrence for the IORT group and the EBRT group. Log-rank test was used to examine the difference between the two survival curves. To investigate the associations between locoregional recurrence and each clinical factor, the hazard ratios (HRs) and 95% CIs for the IORT group compared with the EBRT group were estimated using crude and adjusted Cox proportional hazard models. Subgroup analyses by age or the ASTRO consensus statement risk groups were used to determine any potential differences in response to different adjuvant radiotherapies. In subgroup analysis, unsuitable, and cautionary groups were classified as the non-suitable group for IORT. Statistical analysis was conducted using SPSS for Windows version 21.0 (SPSS Inc., Chicago, IL) software package. A two-tailed *p* value < 0.05 was considered statistically significant.

## Results

### Basic characteristics of the study population

A total of 170 patients were enrolled, with a mean follow-up time of 3.53 ± 0.82 years. The distributions of selected characteristics between the overall 47 patients treated with IORT and the 123 patients treated with EBRT are summarized in Table 1. The average age of the IORT group (55.45 ± 10.52 years) was significantly older than that of the EBRT group (50.02 ± 10.47 years). There were significant differences between the two groups regarding the number of examined lymph nodes (*p* = 0.030). Furthermore, the proportion of N0 stage in the IORT group (100%) was significantly higher than in the RT group (77.24%). There was no significant difference in BMI, cancer type, pT-stage, tumor size, resection margin, ER–PR status, Her-2 status, lymphovascular invasion, chemotherapy, hormone therapy, and target therapy between the two groups.

**Table 1:** Patient demographics and clinical characteristics

	EBRT	IORT	*p* value
Age	50.02 ± 10.47	55.45 ± 10.52	0.005
< 50	59 (47.97)	10 (21.28)	
50–59	41 (33.33)	21 (44.68)	
≥ 60	23 (18.70)	16 (34.04)	
BMI	24.20 ± 3.75	25.03 ± 4.43	0.347
≤ 18	4 (3.25)	1 (2.13)	
18–24	61 (49.59)	18 (38.30)	
> 24	58 (47.15)	28 (59.57)	
Cancer type			0.210
IDC Invasive ductal CA	114 (92.68)	39 (82.98)	
Invasive lobular CA	3 (2.44)	3 (6.38)	
Mucinous CA	5 (4.07)	3 (6.38)	
Papillar CA	1 (0.81)	2 (4.26)	
pT-stage			0.188
T1a	14 (11.38)	3 (6.38)	
T1b	13 (10.57)	8 (17.02)	
T1c	48 (39.02)	25 (53.19)	
T2	47 (38.21)	11 (23.40)	
T3–4	1 (0.81)	0 (0.00)	
Tumor size (mm)			
Mean ± SD	17.18 ± 10.90	15.36 ± 7.41	0.216
Median (IQR)	15 (11–22)	15 (10–19)	
Range	1–60	1–38	
Section margin			0.161
Negative	118 (95.93)	47 (100.00)	
Positive	5 (4.07)	0 (0.00)	
Number of examed lymph node			0.030
1–2	27 (21.95)	18 (38.30)	
3–10	77 (62.60)	27 (57.45)	
> 10	19 (15.45)	2 (4.26)	
pN-stage			0.005
N0	95 (77.24)	47 (100.00)	
N1	23 (18.70)	0 (0.00)	
N2	3 (2.44)	0 (0.00)	
N3	2 (1.63)	0 (0.00)	
ER/PR status			0.281
Negative	20 (16.26)	11 (23.40)	
Positive	103 (83.74)	36 (76.60)	
Her-2 status			0.194
Negative	96 (80.67)	41 (89.13)	
Positive	23 (19.33)	5 (10.87)	
Lymphovascular invasion			0.220
Negative	74 (60.16)	35 (74.47)	
Positive	45 (36.59)	11 (23.40)	
NA	4 (3.25)	1 (2.13)	
Risk group			< 0.001
Suitable	20 (16.26)	17 (36.17)	
Cautionary	48 (39.02)	26 (55.32)	
Unsuitable	55 (44.72)	4 (8.51)	
Chemotherapy			0.082
No	40 (32.52)	22 (46.81)	
Yes (adjuvant)	76 (61.79)	25 (53.19)	
Yes (neoadjuvant)	7 (5.69)	0 (0.00)	
Hormone therapy			0.324
No	23 (18.70)	12 (25.53)	
Yes	100 (81.30)	35 (74.47)	
Target therapy			0.194
No	110 (89.43)	45 (95.74)	
Yes	13 (10.57)	2 (4.26)	
Follow-up time	3.67 ± 0.82	3.18 ± 0.69	< 0.001

### Association between different adjuvant radiotherapies and clinical outcomes

The locoregional recurrence rate was significantly higher in the IORT group than in the EBRT group (5/47, 10.64% vs. 3/123, 2.44%; *p* = 0.024). Four patients in the IORT group had recurrence in the postoperative bed. Three patients in the EBRT group and one patient in the IORT group had a recurrence in the ipsilateral regional lymph nodes. The ipsilateral breast tumor recurrence rate was significantly higher in the IORT group than in the EBRT group (4/5 vs. 0/3; *p* = 0.005). There was no significant difference in distant metastasis, total deaths, and cancer-specific death between the two groups (Table 2).

**Table 2:** Association between different adjuvant radiotherapies and clinical outcomes

	EBRT	IORT	*p* value
Locoregional recurrence			0.024
No	120 (97.56)	42 (89.36)	
Yes	3 (2.44)	5 (10.64)	
Distant metastasis			0.123
No	117 (95.12)	47 (100.00)	
Yes	6 (4.88)	0 (0.00)	
Total deaths			0.379
No	121 (98.37)	47 (100.00)	
Yes	2 (1.63)	0 (0.00)	
Cancer-related death			0.535
No	122 (99.19)	47 (100.00)	
Yes	1 (0.81)	0 (0.00)	
Follow-up time	3.67 ± 0.82	3.18 ± 0.69	< 0.001

### Risk factors of locoregional recurrence

**Fig. 1:**
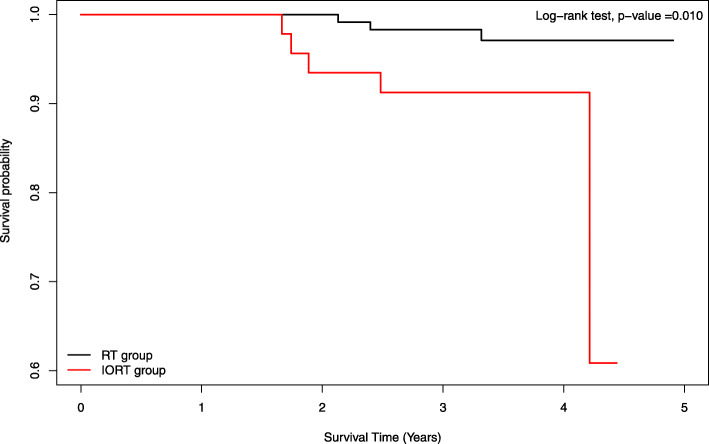
Cumulative incidences of locoregional recurrence in IORT and EBRT groups

The Kaplan–Meier curve of the cumulative probability of locoregional recurrence indicated that the IORT group had a higher risk of locoregional recurrence within 5 years (log-rank test, *p* = 0.010) (Fig. 1). Table 3 shows the Cox regression analysis of risk factors associated with the development of locoregional recurrence. The IORT group had a significantly increased risk of locoregional recurrence compared with the EBRT group after adjustment for clinical and pathologic characteristics (adjusted HR = 52.23; 95% CI = 3.37–809.99; *p* = 0.005). Moreover, there was a significant positive association between the positive resection margin and the risk of locoregional recurrence after adjustment for potential confounders (adjusted HR = 53.91; 95% CI = 3.02–962.98; *p* = 0.007).

**Table 3:** Univariate and multivariate analyses of risk factors for locoregional recurrence

	Crude HR (95% CI)	*p* value	Adjusted HR (95% CI)	*p* value
Radiotherapy type				
EBRT	Reference		Reference	
IORT	5.67 (1.29–24.93)	0.022	52.23 (3.37–809.99)	0.005
BMI				
< 24	Reference		Reference	
≥ 24	1.64 (0.39–6.85)	0.501	1.70 (0.28–10.20)	0.563
Age				
< 50	Reference		Reference	
50–59	0.48 (0.09–2.46)	0.375	0.13 (0.01–1.48)	0.101
≥ 60	0.38 (0.04–3.23)	0.374	0.15 (0.01–1.89)	0.142
Tumor size (mm)	1.00 (0.93–1.08)		1.01 (0.93–1.10)	0.810
Section margin				
Negative	Reference		Reference	
Positive	12.61 (2.44–65.08)	0.002	53.91 (3.02–962.98)	0.007
pN-stage				
N0	Reference		Reference	
N1 + 2	0.93 (0.11–7.71)	0.945	0.38 (0.05–2.73)	0.335
N3	11.32 (1.34–95.33)	0.026	0.28 (0.02–4.84)	0.382
ER/PR status				
Negative	Reference		Reference	
Positive	0.67 (0.14–3.34)	0.629	0.67 (0.08–5.50)	0.713
Her-2 status				
Negative	reference		reference	
Positive	2.96 (0.71–12.40)	0.137	6.06 (0.61–60.46)	0.125
Lymphovascular invasion				
Negative	Reference		Reference	
Positive	1.48 (0.33–6.60)	0.610	5.37 (0.88–32.87)	0.069

### Association between locoregional recurrence and the ASTRO consensus statement risk groups

The frequency distributions of locoregional recurrence among the different ASTRO consensus statement risk groups are shown in Table 4. Among patients in the non-suitable group, compared with those treated with EBRT, those treated with IORT had an HR (95% CI) for locoregional recurrence of 7.02 (1.63–30.16). Among patients younger than 50 years, those treated with IORT had an HR (95% CI) for locoregional recurrence of 10.42 (1.73–62.79) compared with those treated with EBRT.

**Table 4:** Hazard ratios of locoregional recurrence found in the follow-up period associated with the EBRT/IORT group and risk factors

Variables	EBRT			IORT			HR (95% CI)	*p* value
	Event	PY	Rate	Event	PY	Rate		
Suitable	0	73.92	0.00	0	53.01	0.00	NA	
Non-suitable	3	377.33	0.01	5	96.65	0.05	7.02 (1.63–30.16)	0.009
Age < 50	2	218.05	9.17	3	32.04	93.63	10.42 (1.73–62.79)	0.011
Age ≥ 50	1	233.2	4.29	2	117.62	17.00	3.46 (0.31–38.17)	0.311

## Discussion

Our results are generally consistent with the findings of previous studies in that the locoregional recurrence rates were significantly higher with IORT than EBRT (5/47, 10.64% vs. 3/123, 2.44%; *p* = 0.024). Two randomized controlled trials, TARGIT-A and ELIOT, demonstrated a significantly higher local recurrence rate in the IORT group than in the EBRT group. In the ELIOT study, the 5-year local recurrence rate was 4.4% (35/651) in the IORT group and 0.4% (4/654) in the EBRT group (*p* <  0.001) [10]; in the TARGIT-A study, the 5-year local recurrence rate was 3.3% (23/1679) in the IORT group and 1.3% (11/1696) in the EBRT group (*p* = 0.042) [11]. One study conducted in China with a median follow-up of 32 months revealed that the local recurrence rate was 2.78% (2/72) in the IORT group and 1.41% (1/71) in the EBRT group [12]. A retrospective comparison of 4129 patients with pT1N0 breast cancer treated with EBRT (*n* = 2939) and IORT (*n* = 1190) reported a 10-year cumulative risk of locoregional recurrence (axillary relapse) of 1.3% with EBRT versus 4.0% with IORT [13]. Furthermore, in a previous meta-analysis study, ipsilateral breast tumor recurrence was significantly higher in patients with IORT than in those with EBRT (RR, 2.83; 95% CI, 1.23–6.51) [12]. The higher locoregional recurrence risk observed on average in both groups in this study than in other studies might be caused by a higher prevalence of lymphovascular invasion and a smaller sample size (32.9%, 56/170 in this study and 10.8%, 372/3451 in the TARGIT-A study [11]). Although IORT delivers a single large dose to the tumor bed precisely, lack of fractionated radiotherapy to treat occult lesions beyond 1 cm often leads to high locoregional recurrence in long-term follow-up.

As the ASTRO 2016 [9] guidelines are the most recently updated guidelines and are more restrictive than GEC-ESTRO 2010 [14], we adopted the criteria to stratify the patients into different risk groups. None of the patients in the suitable group in the present study had locoregional recurrence irrespective of whether they received IORT or EBRT. In the non-suitable group, patients with IORT alone had a 7-fold greater risk of locoregional recurrence than those with EBRT (HR = 7.02; 95% CI, 1.63–30.16; *p* = 0.009). The ASTRO guidelines note that the use of IORT should be restricted to patients who belong to a suitable group to APBI, based on the Leonardi et al. study, which showed that the guidelines were able to identify the groups for whom IORT might be considered as an effective alternative to EBRT in the ELIOT trial [15]. Our findings also support the idea that low-risk patients identified by the ASTRO 2016 [9] guidelines can undergo IORT without an increased risk of locoregional recurrence, whereas those who do not fulfill the criteria of the suitable group would have a much higher risk of locoregional recurrence if they only received sole IORT. When unexpected final pathologic information, such as resection margin, lymphovascular invasion, tumor size, nodal status, and ER–PR status, predict a higher risk of locoregional recurrence, supplemental EBRT is indicated [3, 11].

Following review of the evidence from three trials [16–18], the age criteria in the ASTRO guidelines for patients suitable for APBI were updated from older than 60 years old in 2009 to 50 years old in 2016. The peak age for breast cancer is between 40 and 50 years in Taiwan, whereas the peak age in Western countries is between 60 and 70 years [6]. As the breast cancer behavior in younger patients tends to be more aggressive [19], whether more restrictive age criteria should be indicated in Taiwan is an interesting topic. In this study, among patients below the age of 50, IORT remained significantly correlated with a 10-fold higher locoregional recurrence risk compared with EBRT but showed a loss of significance in patients over the age of 50. This result implied that the cut-off of 50 years in the ASTRO guidelines was sufficient for patients in our study group. Thus, the eligibility age criteria of 50 years old for IORT for breast cancer might be reasonable according to current evidence. It is important to consider patient age, likely longevity, and the implications of any later increase in locoregional relapse on long-term survival [20]. Age is the only preoperative reliable parameter and should be strictly followed when sole IORT is attempted.

Theoretically, supplemental EBRT was indicated in 64% (30/47) of the IORT group patients in this study because of risky pathological results (20/30) and age under 50 years old (10/30). With a follow-up of 3.18 ± 0.69 years in our study, non-suitable group patients and patients under 50 years old who received IORT alone had approximately 7- and 10-fold greater locoregional recurrence risk than those who received EBRT, respectively. Omission of critical supplemental EBRT in this study reflects a problem in understanding the value and cost of IORT in Taiwan, as IORT costs approximately 8000 USD, which is about half of the average annual personal income. In 2017, the T-IORTSCG study reported a rapid increase in the number of patients who underwent sole IORT, with a locoregional recurrence rate of 0.8% (2/261) over a mean follow-up of 1.3 years [7]. The patients in the T-IORTSCG study tended to be younger (16.5% < 45 years old in T-IORTSCG, 7% < 50 years old in ELIOT, and 2% < 45 years old in TARGIT-A; *p* <  0.01) and have a larger tumor size (T2 tumor percentage: 21.4% in T-IORTSCG, 13% in ELIOT, and 14% in TARGIT-A; *p* <  0.01) than those in the ELIOT and TARGIT-A studies. In the T-IORTSCG study, only 8/261 (3.1%) patients required supplemental EBRT, although 16.5% of them were younger than 45 years old [7]. The expanded inclusion criteria for sole IORT in the T-IORTSCG study and the present study represent the fact that the Taiwanese patients misinterpreted the expensive IORT as a better radiotherapy and were overoptimistic of the preliminary data from aggressive studies [8, 21–25]. Patients are often reliant on their doctors to provide information about the costs of treatment options, considering their individual financial and life plan [26]. The T-IORTSCG study found that young females had a higher motivation to decrease the frequency of hospital visits and were financially more independent to afford the fee of IORT [7]. This may be a reflection of these women having to try harder to balance the needs of family, work, children, and their partners with taking care of themselves [27]. Moreover, a new convenient expensive treatment published in a prestigious journal likely caused the Taiwanese to overlook the impact of unexpected final pathologic information. Jayant S Vaidya et al. emphasized that sole IORT should be used in patients who strictly adhere to the eligibility criteria and suggested that supplemental EBRT should be added [11] to prevent high locoregional recurrence risk in the future whenever higher risk factors exist. Stricter criteria for supplemental EBRT can further mitigate the locoregional recurrence risk as shown by Kristy Broman et al. in 2019 [28]. It is the responsibility of the doctor to support the patients during the process of shared decision making, as well as to convey information relating to the value and limitations of expensive IORT in Taiwan.

Despite higher locoregional recurrence risk for patients who receive IORT than EBRT, there was no significant difference in the risk of distant metastasis, total deaths, and cancer-specific death between the two groups in our study. Results from the SEER database also showed that IORT was not inferior to EBRT when considering the overall survival and cancer-specific death in the short-term follow-up of early breast cancer patients [29]. However, whether locoregional recurrence of breast cancer would impact cancer survival remains unknown. A meta-analysis from the Early Breast Cancer Trialists’ Collaborative Group reported a difference in locoregional relapse rates at 10 years, which may well translate into a survival difference in the longer term [1]. Furthermore, Komoike et al. found that patients with ipsilateral breast tumor recurrence were more likely to develop subsequent distant metastases [30]. Houssami et al. suggested that if all breast cancer recurrences were detected earlier, five to eight deaths would be avoided during a 10-year period for 1000 breast cancer patients (i.e., an absolute reduction in mortality of 17–28%) [31]. In contrast, Sopik et al. concluded that the risk of local recurrence does not correlate with the risk of death from breast cancer across the spectrum of the early stages of breast cancer. After local recurrence, the risk of death from breast cancer depends on the initial stage at diagnosis [32]. Locoregional recurrence might or might not impact breast cancer overall survival, but will definitely impact the patient’s life quality and increase medical expenses. Breast cancer recurrence is a great fear of breast cancer survivors and affects both the patient and their family [33, 34]. Patients considering sole IORT instead of EBRT should be well informed about compatible survival and the higher locoregional recurrence risk, as well as the potential consequences [9].

Except for suitable group patients, a significantly higher locoregional risk was found with IORT than with EBRT over a limited follow-up time. Because of the limitation of the retrospective, unicenter, and non-randomized nature of the present study, as well as the small patient numbers, our results may have higher variability than others. As a result of the lack of intergroup difference, no further detailed subgroup analysis could be conducted, including the impact of lymphovascular invasion, the prevalence of which was doubled compared with the TARGIT-A study [11]. Nevertheless, the results of the present study still support the concept that sole adjuvant IORT should be used under strict the protocols of ASTRO 2016 guidelines for APBI. Negative experiences while practicing beyond the guidelines are also important lessons to learn. Further data collection and longer follow-up is warranted in the future, as well as the inclusion of different races to determine any differences in efficacy.

## Conclusions

A significantly higher locoregional recurrence rate was observed in the IORT group than in the EBRT group. Compared with patients with EBRT, the non-suitable group with IORT alone had around 7-fold greater locoregional recurrence risk, whereas patients under the age of 50 had about 10-fold greater locoregional recurrence risk. Sole adjuvant IORT for breast cancer patients should be administered under strict ASTRO 2016 protocols, and IORT should not be used alone in patients under 50 years old who do not belong to the suitable group.

## Data Availability

The datasets analyzed during the present study are available from the corresponding authors on reasonable request.

## Abbreviations

APBI: Accelerated partial breast irradiation
ASTRO: American Society for Radiation Oncology
BCS: Breast-conserving surgery
CIs: Confidence intervals
EBRT: External beam radiotherapy
HR: Hazard ratio
IORT: Intraoperative radiotherapy
IR: Incidence rate
TARGIT-A trial: Targeted intraoperative radiotherapy versus whole breast radiotherapy for breast cancer
T-IORTSCG: Taiwan Intraoperative Radiotherapy Study Cooperative Group
